# Trajectories of quality of life in people with diabetes mellitus: results from the survey of health, ageing and retirement in Europe

**DOI:** 10.3389/fpsyg.2023.1301530

**Published:** 2024-01-11

**Authors:** Sarah Mendorf, Konstantin G. Heimrich, Hannah M. Mühlhammer, Tino Prell, Aline Schönenberg

**Affiliations:** ^1^Department of Neurology, University Hospital Jena, Jena, Germany; ^2^Department of Geriatrics, University Hospital Jena, Jena, Germany; ^3^Department of Geriatrics, University Hospital Halle, Halle, Germany

**Keywords:** quality of life, diabetes mellitus, longitudinal, older adults, SHARE

## Abstract

**Introduction:**

Previous longitudinal studies identified various factors predicting changes in Quality of Life (QoL) in people with diabetes mellitus (PwDM). However, in these studies, the stability of QoL has not been assessed with respect to individual differences.

**Methods:**

We studied the predictive influence of variables on the development of QoL in PwDM across three waves (2013–2017) from the cross-national panel dataset Survey of Health, Ageing, and Retirement in Europe (SHARE). To determine clinically meaningful changes in QoL, we identified minimal clinically important difference (MCID). Linear regressions and Linear Mixed Models (LMM) were conducted to determine factors associated with changes in QoL.

**Results:**

On average, QoL remained stable across three waves in 2989 PwDM, with a marginal difference only present between the first and last wave. However, when looking at individual trajectories, 19 different longitudinal patterns of QoL were identified across the three time-points, with 38.8% of participants showing stable QoL. Linear regression linked lower QoL to female gender, less education, loneliness, reduced memory function, physical inactivity, reduced health, depression, and mobility limitations. LMM showed that the random effect of ID had the strongest impact on QoL across the three waves, suggesting highly individual QoL patterns.

**Conclusion:**

This study enhances the understanding of the stability of QoL measures, which are often used as primary endpoints in clinical research. We demonstrated that using traditional averaging methods, QoL appears stable on group level. However, our analysis indicated that QoL should be measured on an individual level.

## 1 Introduction

In recent years, the COVID-19 pandemic with its varying policies and circumstances exemplified how quality of life (QoL) can be affected globally and individually by different factors over time (Hansel et al., 2022). For many people, this global stressor caused a reduction in the mental and physical health for a period of time; however, not all people were affected equally. Depending on intrinsic and extrinsic factors, some people maintained a high QoL (Eicher et al., 2021; Malini et al., 2022). These findings raise the question whether QoL can be considered a stable, state-like construct, or whether people are constantly faced with changing circumstances that cause their QoL to oscillate. Notably, QoL is a multi-dimensional and subjective construct that denotes how people view their physical, emotional, and social well-being (Rubin and Peyrot, 1999; Dickerson et al., 2011) and may thus differ for individuals. QoL contributes meaningfully to well-being, and due to its multidimensional approach, people may report high QoL even in the face of physical decline. Still, while QoL exists detached from health, physical health and chronic illnesses are often found to reduce QoL (Samiei Siboni et al., 2019).

One of the most common chronic disorders is Diabetes mellitus (DM), a metabolic disorder characterized by elevated blood sugar levels that affects millions of people worldwide. The disease can lead to a range of complications if not controlled, including neuropathy, visual problems, renal failure, and heart disease (American Diabetes Association, 2013). The diagnosis of DM often requires long-term treatment and changes to lifestyle and dietary habits. These changes can have a significant impact on the quality of life of people with DM (PwDM) (Rubin and Peyrot, 1999). Additionally, for individuals suffering from chronic DM, a comprehensive cure is unattainable (Saleh et al., 2015). A high disease burden due to declining health, worry and medication intake (oral and especially via insulin injections) commonly lead to reduced QoL in PwDM (Yildirim et al., 2023). While clinical measures can offer a helpful assessment of disease management, the primary objective of DM care is to impede deterioration of the patient’s QoL (Saleh et al., 2015). Therefore, identifying the predictors and risk factors impacting QoL is essential, as these can be targeted for preventative measures (Dickerson et al., 2011; Yildirim et al., 2023).

In previous research, several factors that impact the QoL of PwDM have been identified. These include higher age, female gender, higher body mass index (BMI), lower education, more limitations in activities of daily living (ADL), more loneliness, depression and depressive symptoms, higher number of chronic conditions, more mobility restrictions, reduced cognition, widowed or divorced marital status, unemployment or permanent illness at work, less physical activity, reduced general health, and presence of pain (Glasgow et al., 1997; Rubin and Peyrot, 1999; Ij et al., 2012; VanderWeele et al., 2012; Kim and Kim, 2017; Zurita-Cruz et al., 2018; Zakin et al., 2019; Marneras et al., 2020; Al-Matrouk and Al-Sharbati, 2022). Over time, the long-term effects of the disease and its complications can further impact patients’ physical abilities, mobility, social relationships, self-esteem, and mental health (Solli et al., 2010). Disease-specific factors such as stigmatization or the need for insulin injections, as well as complications of the disease progression, may further lead to reduced QoL; resulting in an overall lower QoL of PwDM compared to the general population (Hanestad, 1993; Glasgow et al., 1997; Rubin and Peyrot, 1999; Solli et al., 2010; Ij et al., 2012; VanderWeele et al., 2012; Kim and Kim, 2017; Zurita-Cruz et al., 2018; Zakin et al., 2019; Marneras et al., 2020; Al-Matrouk and Al-Sharbati, 2022; Yildirim et al., 2023). Diabetic neuropathy, for example, has a specific impact on mobility and physical abilities which may lead to difficulty in maintaining social relationships, among other challenges. The condition’s effects on an individual’s movement and coordination are significant. Therefore, its impact must be taken into consideration when developing treatment plans (Solli et al., 2010). Mental wellbeing and self-esteem, alternatively, remains unaffected by glycaemic management and associated complications of DM (Donald et al., 2013). In general, the QoL was significantly affected by ischemic heart disease, stroke, neuropathy, and the fear of hypoglycemia, according to Solli et al. (2010). These complications do not arise at the outset of the illness, only manifesting as the disease advances. Additionally, QoL has emerged as a key objective in healthcare as a result of advancements in patients’ rights movements. Its significance lies in clinical, economic, and political decision-making (Pais-Ribeiro, 2004). This progressive nature of the disease and the accompanying changes in symptomology as well as the aforementioned impact of changes in environmental circumstances, like the COVID-19 pandemic, raise the question of how QoL changes over time in PwDM. Knowing about the temporal development of QoL is crucial in managing the condition and improving overall well-being.

This understanding is especially important as an increasing number of studies utilize QoL as an endpoint in longitudinal analyses. These encompass studies involving PwDM (Schmitz et al., 2013; Wang et al., 2019; Shamshirgaran et al., 2020; Ho et al., 2022; Matlock et al., 2022; Park et al., 2022), as well as other illnesses such as chronic diseases (Le Grande et al., 2006; Dunn et al., 2013; Yoo et al., 2016). It is important to understand the longitudinal trajectories of QoL in PwDM and the factors that impact future QoL. This determines the validity of using QoL as an endpoint in studies (Leidy et al., 1999), because if QoL fluctuates with a high frequency, using it as a one-time endpoint for intervention studies cannot truly uncover the effect of the intervention. Many previous studies addressing report stable QoL for PwDM. For example, a study involving older PwDM discovered that most participants did not experience significant changes of QoL over time. The methods employed in the study consisted of latent class growth analysis and multinomial logistic regression models with the aim of ascertaining the predictors of QoL trajectories. However, this investigation concentrated on the impact of DM on the short-term alterations in the QoL of older adults individuals (Park et al., 2022). It was also discovered in separate studies featuring Taiwanese participants with no age limit that most subjects did not encounter noteworthy QoL modifications over an extended period (Schmitz et al., 2013; Wang et al., 2019) with the use of trajectory modeling (Schmitz et al., 2013; Safita et al., 2016)and multinomial logistic regression (Wang et al., 2019). However, the impact of DM on QoL may also be influenced by cultural variances so that the results cannot be fully transferred to the general population (Safita et al., 2016). Another study looked at older adults PwDM and showed that executive function and negative emotions predicted QoL. Pearson’s correlation coefficient analysis and stepwise multiple linear regression analysis have been conducted. The study’s limitation lies in its inadequate sample size, which precludes confirming the findings amongst various older adults diagnosed with DM and having numerous variables (Ho et al., 2022). Shamshirgaran et al. compared individuals with and without DM and concluded that their QoL increased over time. Age, chronic conditions, vision and sleep problems, body weight, annual income, and years of education were found to be predictive factors for future QoL. The study employed the Generalised Estimating Equation model to achieve its objective. Caution should be taken when considering the generalizability of the results due to the participation rate, dropouts, and loss to follow up (Shamshirgaran et al., 2020). While these results assessed the stability of QoL while controlling for covariates, it is important to note that these studies assessed QoL on group-average instead of considering individual patterns of QoL across time. However, to truly understand the reliability of QoL as an endpoint for clinical trials, it is crucial to separate the average effect from individual influences. This is because, a stable mean or consistent QoL may also imply that different patterns within the cohort cancel each other out.

The aims of this study are therefore to longitudinally depict the patterns of QoL in PwDM and to estimate the effects of variables that vary between individuals as well as variables that vary within individuals.

## 2 Methods

### 2.1 Study design and population

This study utilized data from the Survey of Health, Ageing and Retirement in Europe (SHARE). This is a large multi-national panel dataset, encompassing over 140,000 subjects aged 50 and above from 20 European nations and Israel. SHARE represents a continual research partnership exploring the influence of health, social, economic, and environmental policies on the lives of middle-aged and older adults individuals throughout Europe. Currently, eight waves of SHARE data have been gathered with both new and repeating participants to enable cross-sectional and longitudinal data analysis of a wide range of variables. Skilled staff in each country collect these variables using a computer-assisted personal interviewing method, occasionally supported by distributing paper-pencil questionnaires. Furthermore, individuals were summoned to designated study centres to undertake a range of health evaluations, including tests for cognitive ability, grip efficacy, walking pace as well as blood sampling (SHARE, n.d.). In the development stage of the sampling process, the Country Team and Field Agency work alongside SHARE Central to craft a tailored sampling design. This involves deciding on a sampling frame and specifying the sampling procedure. Secondly, the sample is drawn by the Country Team / Field Agency (or through a third party, such as an institution hosting a national register) and then processed to produce a gross sample file. In the third stage, SHARE Central ensures the conformity of the gross sample with SHARE standards. In the final step, SHARE Central and CentERdata collate and upload the gross sample data using Sample Control software, which is subsequently merged with the corresponding addresses provided by the Country Team. The detail survey methods, sampling design, and data resources are described in detail in the respective survey materials (Börsch-Supan et al., 2013a,b; Malter and Börsch-Supan, 2013; Börsch-Supan et al., 2015; Malter and Börsch-Supan, 2015; Malter and Börsch-Supan, 2017). Due to probabilistic sampling the participants selected were nationally representative. The survey questions pertained to measures of demographics, socio-relational factors, as well as health-related indices, encompassing functional ability and mental health. Our data for this investigation has been extracted from waves 5 (2013) to 7 (2017) (Börsch-Supan, 2022a,b,c) of the SHARE dataset.

The inclusion criteria were the presence of DM and a complete CASP score without missing data. Due to the careful sampling procedure employed in SHARE and the exploratory nature of our analysis, no further exclusion criteria were specified.

### 2.2 Measures

For ease of understanding and replicability, we provide the name of the variables as given in the SHARE dataset in italics and the name of the respective dataset in square brackets in Table 1 (Börsch-Supan, 2022a,b,c).

**Table 1:** Overview of independent variables.

Variable (*SHARE dataset denomination)*	Derived from dataset…	Value
Sex *(gender)*	General variables dataset [gv_imputations]	Female = 1, male = 0
Age *(age)*	General variables dataset [gv_imputations]	In years
BMI *(bmi)*	General variables dataset [gv_imputations]	In kg/m2
Number of chronic diseases *(chronic)*	General variables dataset [gv_imputations]	
Marital status *(mstat)*	General variables dataset [gv_imputations]	answer options: None Married and living together with spouse, Registered partnership, married, Living separated from spouse, Never married, Divorced, Widowed.
Current job situation *(cjs)*	General variables dataset [gv_imputations]	“In general, how would you describe your current situation?”: retired, Employed or self-employed (including working for family business), Unemployed, Permanently sick or disabled, Homemaker
Limitations of ADL *(adl)*	General variables dataset [gv_imputations]	Participants check off which activities they have difficulty with. The number was summed up. The following ADLs encompassed dressing, including putting on shoes and socks; walking across a room; bathing or showering; eating, such as cutting up your food; getting in or out of bed; and using the toilet, including getting up or down. The higher the index is the more difficulties with these activities and the lower the mobility of the respondent. *Adl* ranges from 0 to 6.
Education *(yedu)*	General variables dataset [gv_imputations]	In years; asking how long the participant had been in full-time schooling and vocational training.
Self-rated memory function *(memory)*	General variables dataset [gv_imputations]	Asking participants how they rated their memory function using a 5-point Likert scale (excellent, very good, good, fair, and poor).
Self-perceived/rated health *(*SRH, *sphus)*	General variables dataset [gv_imputations]	5-point Likert scale (excellent, very good, good, fair, and poor).
Depressive symptomology measured by the Centre for EURO-D	General variables dataset [gv_imputations]	This scale comprises 12 binary items related to the subsequent symptoms: sadness, pessimism, thoughts of suicide, guilt, sleep disturbances, diminished interest in activities, irritability, reduced appetite, fatigue, impaired concentration, lack of enjoyment, and tearfulness. Each item is assigned a score of either 0 or 1, with 1 consistently denoting a negative emotional state (i.e., 1 = higher degree of depression). The scores for all items are then aggregated, resulting in a total score ranging from 0 to 12 (Prince et al., 1999). Cronbach’s alpha is 0.83 (Tomás et al., 2022).
Loneliness *(loneliness)*	General variables dataset [gv_health]	Measured with the short version (Hughes et al., 2004) of the R-UCLA Loneliness Scale (revised UCLA) (Russell et al., 1978). It assesses indirect feelings of loneliness. The three items – companionship, feeling left out, and isolation – are rated using a three-point Likert scale (“often,” “occasionally,” “rarely or never”). The calculated score ranges from a minimum of 3 (“not experiencing loneliness”) to a maximum of 9 (“feeling very lonely”). Cronbach’s alpha is 0.73 (Russell, 1996).
Mobility limitations *(mobility)*	General variables dataset [gv_imputations]	Participants check off which activities they have difficulty with. The number was summed up. The following mobility criteria were asked about: walking 100 meters, walking across a room, climbing several flights of stairs, and climbing one flight of stairs. The higher the index, the more difficulties exist and the lower the mobility of the respondent. It ranges from 0 to 4.
Physical inactivity *(phinact)*	general variables dataset [gv_imputations]	Asking participants if they are physical inactive (yes/no).
Pain *(ph085_)*	Physical health dataset [ph]	“Are you troubled with pain?” (yes/no).

For ease of understanding and replicability, this table presents the variable names given in the current manuscript as well as the original variable name in the SHARE dataset and the dataset of origin, as SHARE is comprised of multiple datasets for varying domains (Tomás et al., 2022).

The presence of DM was captured by a yes/no question: “Has a doctor ever told you that you had Diabetes or high blood sugar?” (*ph006d5 in physical health dataset [ph]*). No distinction was made between type 1 and type 2 DM.

#### 2.2.1 Dependent variable:

The CASP-12 score (*casp in general variables dataset [gv_health]*) evaluates the QoL and is determined by four subdimensions: control, autonomy, pleasure, and self-realization. These four subdomains also gave the score its name: CASP: control, autonomy, self-realization, and pleasure. This measure distinguishes itself from health-related measures by placing emphasis on the positive aspects of QoL and by operating independently from health and other external factors that could potentially impact on it (Hyde et al., 2003; Wiggins et al., 2008). One’s capability to shape their environment is known as control, while the accompanying need for self-determination is called autonomy, both of which are essential for full participation within society (Hyde et al., 2003). Self-realization and pleasure domains pertain to the level of fulfilment of human potential and hedonic well-being, respectively (Higgs et al., 2003). As the survey is intended for older adults individuals (Hyde et al., 2003), it highlights crucial elements of the QoL for those with DM, particularly DM Type ll, which is a pervasive condition affecting the older adults populace.

The CASP score is computed by summing up these four subdimensions, resulting in a range of 12 to 48. A high score indicates high QoL (Hyde et al., 2003). The Cronbach’s alpha values for the domain control, autonomy, pleasure, and self-realization are 0.73, 0.33, 0.74, and 0.85, respectively (Borrat-Besson et al., 2015).

In total, 3,985 PwDM had a complete CASP in wave 5, 4,366 participants in wave 6, and 4,643 in wave 7. Across all waves, 2,989 PwDM with a complete CASP were identified. These participants were included in the longitudinal analyses presented in our manuscript.

#### 2.2.2 Independent variables

Due to the complex nature of the dataset, the extracted variables are presented in detail in Table 1.

### 2.3 Statistical analysis

All analyses were conducted using IBM SPSS statistics (Version 29) and R (Version 4.1.1). The included parameters were not normally distributed, as assessed by the Shapiro–Wilk-Test. For this reason, metric values are given as median with interquartile range (IQR). Nominal and ordinal values are given as numbers and percentages. Missing data were treated according to the pairwise deletion process. All statistical tests were applied two-sided at a significance level of 0.05.

#### 2.3.1 Comparison between the waves

To understand the nature of our dataset and interpret the results of the longitudinal analyses, we first describe the variables of interest for each wave. Then, we used Friedman test for ordinal und metric variables and Cochrane’s Q for nominal variables with *post hoc* analysis adjusted by Bonferroni correction to detect changes in the variables of interest between the three waves. Of note, as the Friedman test is based on ranks and not the actual (median) values, considerable changes can be discovered despite the medians seeming to be identical (Friedman, 1937).

#### 2.3.2 Cross-sectional analysis of potential factors influencing QoL in each wave

A general linear model for each wave (stepwise backward) for the CASP score was calculated to identify variables that influence QoL in PwDM cross-sectionally. According to a Durbin Watson Test of >1.5 and < 2, autocorrelation was not an issue in the model. Evidence for multicolinearity did not emerge for Variance inflation factor values between 1 and 2 in all regressions of the respective waves.

#### 2.3.3 Calculation of the minimum clinically important difference

As the CASP score can encompass up to 48 points, we aimed to control for random fluctuations in QoL scores across time points. Therefore, as a first step, we aimed to determine when a change in the CASP score across waves could be considered a meaningful/impactful change. Utilizing any change, starting with a single point, is not feasible due to measurement errors and memory bias. Instead, we chose the minimal clinically important difference (MCID) as the clinically significant difference, taking into consideration the standard deviation (SD) of CASP scores.

We calculated this using the distribution method: MCID = 0.5 * SD (Norman et al., 2003, 2004).

In our case, the formula was:

$$\mathrm{MCID}=0.5^{*}\;\left[\left(\begin{array}{l} {SD}_{\mathrm{wave}5}+\\ {SD}_{\mathrm{wave}6}+\\ {SD}_{\mathrm{wave}7} \end{array}\right)/3\right]=0.5^{*}\;\left[\left(\begin{array}{l} 6.25+\\ 6.34+\\ 6.47 \end{array}\right)/3\right]=3.18\text{.}$$

The MCID was 3.18; therefore, we considered CASP-12 differences between waves of ≥4 to be clinically significant. Thus, based on the MCID, we divided the participants into 2 main groups: stable QoL across all three waves, and unstable/changing QoL across the waves. Stable QoL indicates that participants’ CASP scored did not differ more than 3 points between the waves, unstable QoL means that the CASP score did differ ≥4 points between at least two waves.

The MCID has been defined as the smallest score difference in the relevant score that patients perceive as advantageous and that would demand a change in the patient’s treatment while ensuring there are no severe side effects or exorbitant costs (Jaeschke et al., 1989). Osoba et al. (1998) highlight the significance of identifying clinically meaningful differences, since even minor numerical disparities in mean health-related QoL scores may yield statistically significant results with generous sample sizes, but statistical significance is not interchangeable with clinical significance. One advantage of distribution-based methods is their ability to account for changes beyond a certain level of random variation (Crosby et al., 2003). Distribution-based methods are specific to the sample used. For instance, statistical analysis alone can extract MCID scores from a study with a large sample size and wide distribution, even if no actual change has occurred (Wright et al., 2012). However, distribution-based methods have a weakness in that there are few established benchmarks for determining clinically significant improvements. More significantly, distribution-based methods are inadequate in addressing the question of a patient’s perspective of clinically important change, which varies distinctly from statistical significance (Crosby et al., 2003; Copay et al., 2007).

#### 2.3.4 Patterns of QoL change across the waves

Having determined at which point a change in the CASP score reflects a meaningful change in QoL, we first calculated the differences between the CASP sum scores for Wave 5 – Wave 6, Wave 6 – Wave 7 and Wave 5 – Wave 7 for every participant. To descriptively assess the different patterns in QoL, differences ≥ ±4 were denoted as ±1 (depicting a clinically relevant increase or decrease between the respective waves), while differences below 4 were coded as 0. We then assessed the number of distinct patterns of increase, decrease and stability for the longitudinal subsample and provide graphic illustrations of those patterns.

### 2.4 Longitudinal analysis of predictors of QoL using linear mixed models

Finally, to determine which variables drive this change in QoL, a linear mixed model (LMM) was employed to examine the relationship between CASP scores and (1) only the waves as fixed intercept, and (2) when adding the covariates waves, age, limitations of ADL, number of chronic illnesses, education level, depressive symptoms, SRH, loneliness, job status, marital status, memory, mobility limitations, pain, and physical inactivity as fixed effects predictors. ID was used as random effect predictor. Random intercepts for participants were specified to account for the repeated measures nature of the data. Model fit was assessed using Akaike’s information Criterion (AIC), and the significance of the fixed effects was determined based on *p*-values. Additionally, we calculate the Intraclass Correlation Coefficient (ICC) using the formula:

$$\frac{\mathrm{variance}\;\mathrm{of}\;\mathrm{random}\;\mathrm{effect}\;}{\left(\mathrm{variance}\;\mathrm{of}\;\mathrm{random}\;\mathrm{effect}+\mathrm{variance}\;\mathrm{of}\;\mathrm{residual}\right)}$$

## 3 Results

The sociodemographic information on the included participants is given in Table 2. Among the waves, the proportion of women ranged between 50.9 and 51.7%. The median CASP score across waves was 37 (IQR 32–41), suggesting – across the entire cohort – a stable QoL over time. Education in years (*p* = 0.215), gender (*p* = 1.000), memory function (*p* = 0.819), and pain (*p* = 0.154) showed no significant change between waves (Table 2). The median age incrementally increased by 2 years from wave to wave according to the time course of the waves. Furthermore, the percentage of married PwDM decreased while widowhood became more prominent. On average, the BMI decreased significantly between waves 5–6 and 6–7, while ADL limitations increased between waves 5–7. Of note, CASP scores decreased significantly between waves 5–7, but not between waves 5–6. In contrast, R-UCLA, EURO, and the proportion of retired PwDM significant increased both from waves 5–7 and from waves 6–7. Correspondingly, the percentage of PwDM in employment, unemployment or long-term sickness decreased. Furthermore, there was a decline in verbal fluency, as well as a decrease in the percentage of participants reporting improved SRH and physical activity between waves 5/7 and waves 6/7. In contrast, there was an increase in reduced SRH and physical inactivity during the same period. The number of chronic illnesses and mobility limitations increased considerably between all waves (Table 2).

**Table 2:** Demographic characteristics and comparison between the waves for PwDM.

Variable	Wave 5 (*n* = 3,985)	Wave 6 (*n* = 4,366)	Wave 7 (*n* = 4,643)	Wave comparison	Post-hoc analysis		
	Median (IQR)	Median (IQR)	Median (IQR)		5–6	5–7	6–7
Age in years	68 (62–75)	70 (64–76)	72 (66–78)	**<0.001**	**<0.001**	**<0.001**	**<0.001**
BMI in kg/m2	29 (26–33)	29 (26–33)	29 (26–33)	**<0.001**	0.376	**<0.001**	**<0.001**
Education	11 (8–13)	11 (8–13)	11 (8–13)	0.215	0.821	0.928	0.751
Limitations in ADL	0 (0)	0 (0)	0 (0)	**<0.001**	0.183	**0.001**	0.056
CASP	37 (32–41)	37 (32–41)	37 (32–41)	**0.011**	0.362	**0.005**	0.055
R-UCLA	3 (3–4)	3 (3–5)	3 (3–5)	**<0.001**	0.185	**<0.001**	**0.042**
EURO	2 (1–4)	2 (1–4)	2 (1–4)	**<0.001**	0.720	**<0.001**	**<0.001**
Number of chronic deseases	3 (2–4)	3 (2–4)	3 (2–4)	**<0.001**	**0.004**	**<0.001**	**<0.001**
Mobility limitations	1 (0–4)	2 (0–4)	2 (0–4)	**<0.001**	**0.011**	**<0.001**	**<0.001**
Verbal fluency	19 (15–25)	19 (15–25)	18 (13–23)	**0.003**	0.429	**0.019**	**0.002**

		*n* (%)	*n* (%)	*n* (%)	Wave comparison	Post-hoc analysis		
Sex	Male	1924 (48.3%)	2,113 (48.4%)	2,281 (49.1%)	1.000	1.000	1.000	1.000
	Female	2061 (51.7%)	2,253 (51.6%)	2,362 (50.9%)				
	Missing	0	0	0				
Marital status	Married, living with spouse	2,672 (67.1%)	2,855 (65.4%)	2,948 (63.5%)	**<0.001**	0.183	**0.006**	0.159
	Registered partnership	56 (1.4%)	60 (1.4%)	51 (1.1%)				
	Married, not living with spouse	62 (1.6%)	62 (1.4%)	61 (1.3%)				
	Never married	207 (5.2%)	230 (5.3%)	248 (5.3%)				
	Divorced	328 (8.2%)	370 (8.5%)	394 (8.5%)				
	Widowed	660 (16.6%)	789 (18.1%)	941 (20.3%)				
	Missing	0	0	0				
SRH	Excellent	75 (1.9%)	85 (1.9%)	65 (1.4%)	**<0.001**	0.505	**<0.001**	**<0.001**
	Very good	325 (8.2%)	352 (8.1%)	333 (7.2%)				
	Good	1,335 (33.5%)	1,446 (33.1%)	1,437 (30.9%)				
	Fair	1,592 (39.9%)	1764 (40.4%)	1955 (42.1%)				
	Poor	658 (16.5%)	719 (16.5%)	853 (18.4%)				
	Missing	0	0	0				
Job situation	Retired	2,706 (68.3%)	3,139 (72.4%)	3,466 (75.5%)	**<0.001**	0.062	**<0.001**	**0.025**
	Employed or self-employed	591 (14.9%)	580 (13.4%)	528 (11.5%)				
	Unemployed	122 (3.1%)	80 (1.8%)	63 (1.4%)				
	Permanently sick	188 (4.7%)	160 (3.7%)	156 (3.4%)				
	Homemaker	310 (7.8%)	312 (7.2%)	307 (6.7%)				
	Other	45 (1.1%)	67 (1.5%)	73 (1.6%)				
	Missing	23 (0.6%)	28 (0.6%)	50 (1.1%)				
Memory function	Excellent	223 (5.6%)	240 (5.5%)	69 (5.4%)	0.819	0.941	0.970	0.970
	Very good	646 (16.2%)	715 (16.4%)	190 (15.0%)				
	Good	1719 (43.1%)	1897 (43.4%)	552 (43.6%)				
	Fair	1,126 (28.3%)	1,236 (28.3%)	373 (29.4%)				
	Poor	271 (6.8%)	278 (6.4%)	83 (6.6%)				
	Missing	0	0	3,376 (72.7%)				
Physical inactivity	No	3,414 (85.7%)	3,695 (84.6%)	1,009 (79.6%)	**<0.001**	0.950	**<0.001**	**0.004**
	Yes	571 (14.3%)	671 (15.4%)	258 (20.4%)				
	Missing	0	0	3,376 (72.7%)				
Pain	Yes	2,140 (53.7%)	2,460 (56.3%)	744 (58.7%)	0.154	0.889	0.082	0.110
	No	1845 (46.3%)	1906 (43.7%)	523 (41.3%)				
	Missing	0	0	3,376 (72.7%)				

IQR, Interquartile range; BMI, body mass index; ADL, activites of daily living; CASP, control, autonomy, self-realization, and pleasure QoL score; EURO-D, depressive symptoms questionnaire; R-UCLA, Revised UCLA Loneliness Scale; SRH, self-rated health. Values highlighted in bold indicate significant results.

### 3.1 Factors cross-sectionally associated with QoL

In the linear regression, we identified a set of variables to be associated with lower QoL across all three waves. These include female gender, less years of education, loneliness, reduced memory function, physical inactivity, reduced SRH, depressive symptomology, and more mobility limitations. Pain and a higher number of chronic diseases were additionally associated with poorer QoL in PwDM only in wave 6 (Supplementary Table S1).

### 3.2 Development of QoL

**Figure 1:**
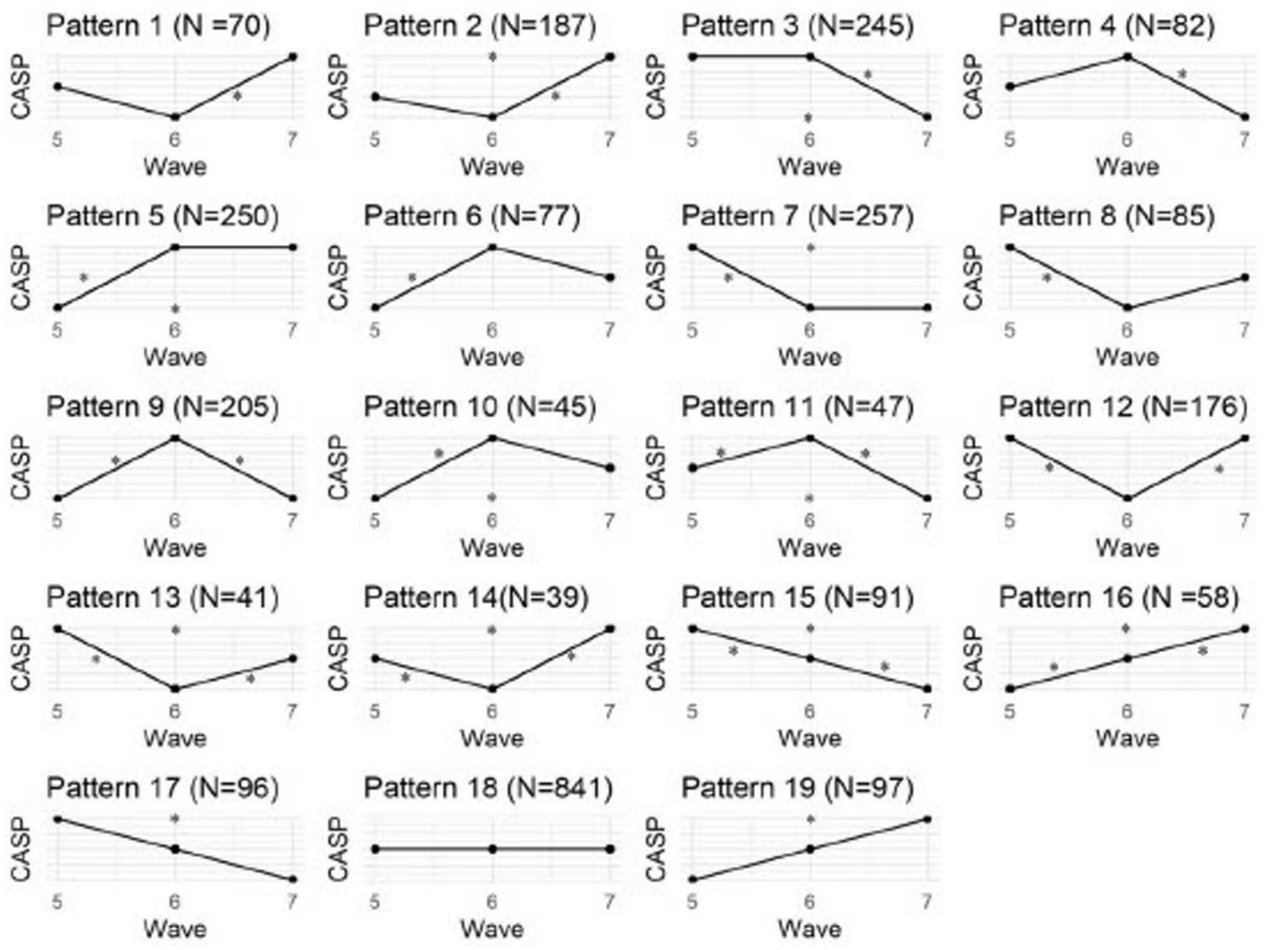
CASP pattern. * indicate differences of points in the CASP between waves, N indicates the number of participants showing the respective pattern.

Having confirmed the link between QoL and several variables cross-sectionally, we next aimed to assess their longitudinal influence on QoL. For this purpose, we selected a sub-sample of 2,989 PwDM who completed the CASP in all three waves. Sociodemographic information on this sub-group is given in Supplementary Table S2. We used a MCID of ≥4 points as a relevant difference in CASP score between waves. Overall, 61.2% (*n* = 2,148) showed a relevant change in QoL across all 3 waves (unstable QoL). When analyzing possible structures behind these CASP changes, 19 patterns emerged (Figure 1). These patterns depict changes (increase or decrease) between wave 5–6, waves 6–7 as well as waves 5–7 that exceed the identified MCID. Overall, the patterns indicate a varying trajectory of QoL across three time-points, with the most common patterns being a stable CASP score (pattern 18, *N* = 841), a decrease from wave 5 to 7 but stable QoL between 6 and 7 (pattern 7, *N* = 257), an increase between wave 5 and 7 but no change between 6 and 7 (pattern 5, *N* = 250), and a decrease between wave 5 and 7 but no change between 5 and 6 (pattern 3, *N* = 245). Using group comparison, we identified that those with unstable QoL had significantly less education, more ADL limitations, more loneliness, and depressive symptoms, more chronic conditions, more mobility limitations, lower SRH, reduced memory function, increased physical inactivity, and other job situations. However, the effect sizes were very small (Supplementary Table S2).

### 3.3 Linear mixed models

To fully understand what drives the differences in CASP cores across waves, we used LMMs. Initially, we aimed to differentiate between group effects and individual effects, as the different patterns suggest a highly heterogenous trajectory of QoL across the three waves. Thus, we performed initial simple model (Table 3) containing the respective *CASP* score of each wave as the dependent variable as well as *wave* as a fixed and *ID* as a random effect. An ANOVA showed that the null-hypothesis that the CASP does not change between waves has to be rejected, *F* (2, 5,978) = 6.197, *p* = 0.002), meaning that the CASP varies between waves. As already indicated in the descriptive results, this effect is mainly driven by the difference between W5 and W7 (*p* = 0.002). When looking at the results in the form of a simple regression model using model slope and intercept,

$$f\;\left(x\right)=36.344:0.003^{*}\;{QoL}_{\mathrm{wave}\;0.6}:0.292^{*}\;{QoL}_{\mathrm{wave}7}\text{,}$$

**Table 3:** Simple LMM with Wave and ID.

	CASP		
*Predictors*	*Estimates*	*CI*	*p*
(Intercept)	36.34	36.11–36.57	**<0.001**
Wave 6	−0.00	−0.19 – 0.18	0.972
Wave 7	−0.29	−0.48 – −0.11	**0.002**
*Random effects*			
σ^2^	13.53		
τ_00 id_	27.44		
ICC	0.67		
N _id_	2,989		
Observations	8,967		
Marginal R^2^ / Conditional R^2^	0.000 / 0.670		

CASP, control, autonomy, self-realization, and pleasure QoL score, CI, Confidence interval, ICC, Intraclass Correlation Coefficient. Values highlighted in bold indicate significant results.

The results imply that on average, QoL reduces by 0.003 points between wave 5 and wave 6, and by 0.292 points from wave 5 to wave 7. In line with the descriptive results in Table 2, this also suggests that, on group level, the QoL remains relatively constant.

However, the random effect also showed a strong effect of ID on the CASP. Looking at the explained variance indicates a variance of 24.47 for the random effect of ID with only 37.997 residual variance. We calculate the ICC revealing high intra-personal clustering with an ICC of 0.67, meaning that 67% of the model variance can be explained by the ID alone.

**Figure 2:**
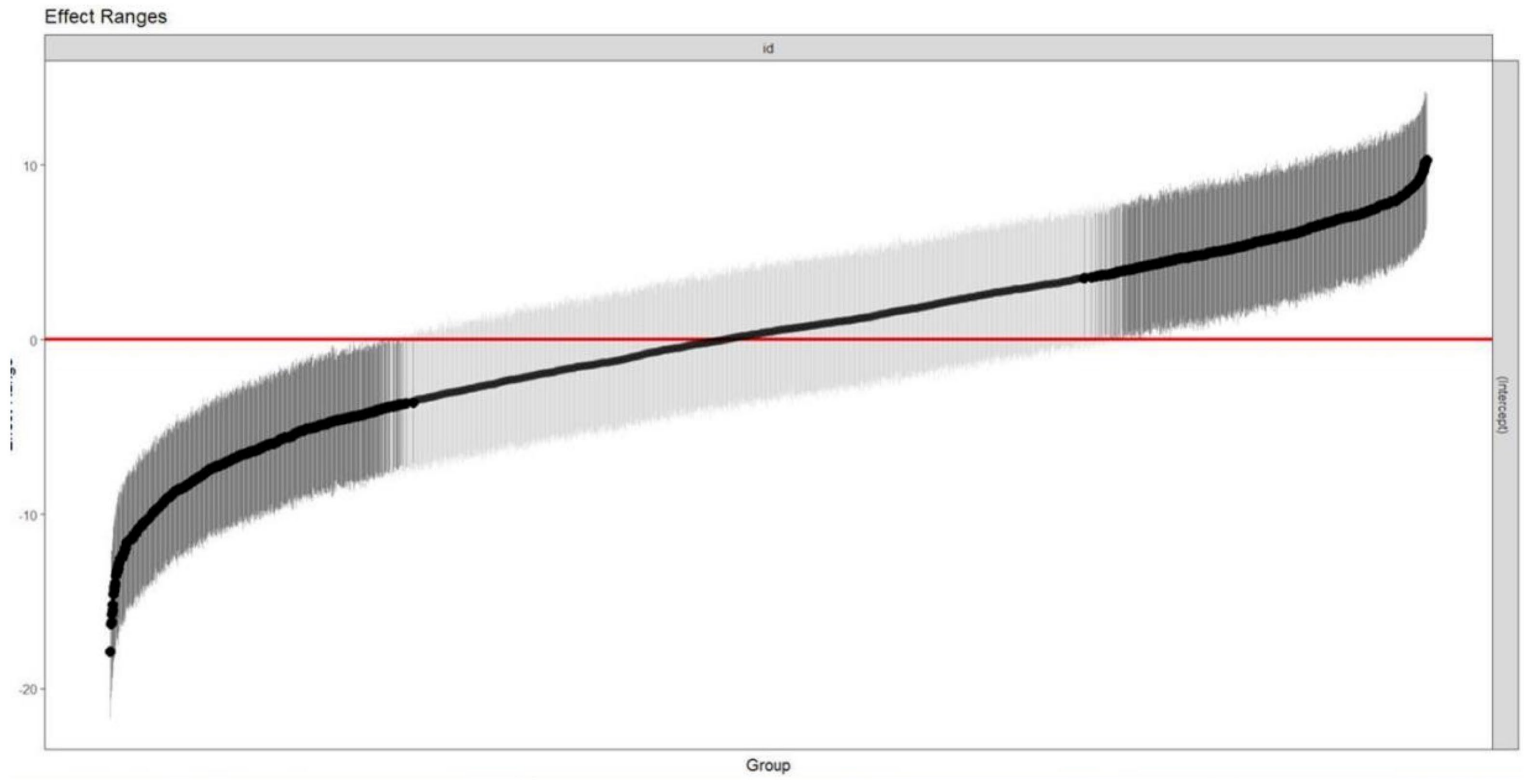
Plot of the estimated random effects for each ID and their Confidence Intervels.

Figure 2 shows the estimated random effects (intercept) for each ID and their confidence interval estimate. Intervals that do not include zero are in bold. In this case, such IDs are relatively higher or lower starting out compared to a “typical” ID. These results show the high range of random effects, again indicating that on an individual level, CASP scores vary between the waves.

To ultimately differentiate between the influence of ID and the covariates previously identified, we calculated a second linear mixed model with CASP as dependent variable and several covariates as fixed effects (Table 4). Model comparison using ANOVA revealed a better fit for the model containing covariates vs. the initial simple model (*p* < 0.001), indicating that the added covariates contribute to understanding the changes in CASP. When looking at the covariates, a significant negative effect on QoL can be found for depressive symptoms (EURO-D), education, number of chronic illnesses, SRH, loneliness, cognition, mobility, physical inactivity, and female gender. However, the random effect of ID still showed an ICC of 0.463, and conditional R^2^ was higher than marginal R^2^, indicating a strong influence of ID on the CASP scores. Marginal R^2^ depicts the variance explained by fixed factors only, whereas conditional R^2^ takes into consideration the variance explained by fixed and random effects.

**Table 4:** LMM with covariates.

	*CASP*		
*Predictors*	*Estimates*	*CI*	*p*
(Intercept)	44.91	43.18–46.65	**<0.001**
Wave 6	0.13	−0.05 – 0.30	0.152
Wave 7	0.12	−0.17 – 0.42	0.414
Age	−0.01	−0.03 – 0.01	0.168
Limitations in ADL	−0.06	−0.21 – 0.09	0.433
EURO-D	−0.72	−0.78 – −0.66	**<0.001**
BMI	−0.02	−0.04 – 0.01	0.165
Chronic diseases	−0.11	−0.19 – −0.03	**0.006**
Education	0.07	0.03–0.10	**<0.001**
SRH - Excellent	−2.52	−3.10 – −1.93	**<0.001**
SRH - Very Good	−0.33	−0.79 – 0.13	0.157
SRH - Good	−0.17	−0.52 – 0.18	0.354
SRH - Fair	0.07	−0.17 – 0.31	0.555
R-UCLA	−0.90	−0.98 – −0.81	**<0.001**
Marital status	0.04	−0.03 – 0.11	0.273
Memory - Very Good	−0.45	−1.14 – 0.24	0.199
Memory - Good	−1.19	−1.83 – −0.56	**<0.001**
Memory - Fair	−1.83	−2.49 – −1.16	**<0.001**
Memory - Poor	−3.63	−4.47 – −2.80	**<0.001**
Mobility	−0.26	−0.33 – −0.20	**<0.001**
Pain - No	0.36	0.12–0.61	**0.004**
Sex - Female	0.38	0.08–0.68	**0.012**
Physical inactivity - Yes	−0.76	−1.09 – −0.44	**<0.001**
*Random effects*			
σ^2^	11.51		
τ_00 id_	9.91		
ICC	0.46		
N _id_	2,989		
Observations	6,783		
Marginal R^2^ / Conditional R^2^	0.439 / 0.699		

BMI, body mass index; ADL, activites of daily living; CASP, control, autonomy, self-realization, and pleasure QoL score; EURO-D, depressive symptoms questionnaire; R-UCLA, Revised UCLA Loneliness Scale; SRH, self-rated health; CI, Confidence Interval; ICC, Intraclass Correlation Coefficient. Values highlighted in bold indicate significant results.

## 4 Discussion

When researching QoL as an endpoint, it is crucial to appreciate PwDM’s QoL progression over time, as well as the factors influencing it. Our findings demonstrate a relatively constant QoL for PwDM when measured in the aggregated way using traditional group comparison or regression methods. This was also confirmed in other studies, which showed that PwDM had a relatively stable QoL (Schmitz et al., 2013; Wang et al., 2019; Park et al., 2022). However, when looking at the present patterns of QoL changes, stable QoL based on a MCID of 4 was only found in 38.8% PwDM. Our results of the LMMs and the different patterns presented in Figure 2 suggest that this is likely a result of individual developments balancing each other out when considered on group level. This means that the QoL increase in some and the decrease in others cancel each other out, leading to a deceiving stability of QoL on group level. However, there is considerable variability at the individual level, suggesting that individuals’ QoL is more influenced by their own circumstances than by other medical, social, and psychological factors. This indicates that in line with the idea that QoL describes the personal interpretation of various mental, health-related and social life circumstances (Samiei Siboni et al., 2019), QoL in PwDM is highly individual. This has also been demonstrated in older adults individuals irrespective of their pre-existing medical conditions (Bowen et al., 2015; Howard and Hoffman, 2018; Băjenaru et al., 2022). This is attributed to differing socio-demographic characteristics, needs, and perceptions towards health, resulting in non-uniform profiles (Balog et al., 2020). In general, there is notable variation in the individual health and QoL (Howard and Hoffman, 2018).

In addition to individual influences, we did identify common factors that explain variability in QoL. The connection between chronic conditions and QoL for PwDM is in line with previous research, given that the physical health status, including complications like diabetic retinopathy, neuropathy, and nephropathy, can reduce QoL (Bowen et al., 2015; Matlock et al., 2022). SRH, which has been found to contribute to QoL (Ferrans et al., 2005), was also identified as a significant forecaster of QoL in this analysis, which is in line with previous studies in which older PwDM who reported better health also had higher QoL than their counterparts (Schmitz et al., 2013; Shiu et al., 2014; Speight et al., 2020). Park et al. (2022) found that PwDM who have higher levels of education, physical activity, and are male were more likely to be classified in the stable or increasing QoL groups which is also reflected in our results. Other studies have also demonstrated the predictive influence of pain, executive function-related memory (Ho et al., 2022), and education (Shamshirgaran et al., 2020). Additionally, individuals with other chronic illnesses also demonstrate a relationship between depression and QoL (Le Grande et al., 2006; Yoo et al., 2016). In General, depression or depression-related symptoms are commonly associated with QoL (Malhi and Mann, 2018). The results emphasize the complex interplay between physical, psychological, and social variables among PwDM and other illnesses that constitute QoL as a personal interpretation of different life domains (Samiei Siboni et al., 2019). Including these health-related and psychosocial variables in our analysis is important, as this allows for an estimation of the life domains that contribute to variations in QoL on the one hand, and to compare the influence of individual fluctuations to common changes during chronic illness. Likewise, these results show that our study population is comparable to previous populations on the general level. Still, the comparison of the marginal to the conditional R^2^ representing fixed and random effects indicates that the contribution of the covariates is comparably low when considering the individual.

The factors that consistently had a negative impact on QoL, based on cross-sectional analysis, were female gender, lower levels of education, loneliness, reduced memory function, physical inactivity, poorer SRH, depressive symptoms, and greater mobility limitations. It is worth noting that these factors have been reported in previous studies (Verbrugge, 1982; Hibbard and Pope, 1983; Verbrugge, 1985; Green, 1990; Kandrack et al., 1991; Sharpe et al., 1991; Jacobson et al., 1994; Glasgow et al., 1997; Jacobson et al., 1997; Black, 1999; Rubin and Peyrot, 1999; Pouwer et al., 2005; Hermanns et al., 2006; Ij et al., 2012; VanderWeele et al., 2012; Kim and Kim, 2017; Zurita-Cruz et al., 2018; Zakin et al., 2019; Marneras et al., 2020; Al-Matrouk and Al-Sharbati, 2022). Additionally, education level was related to QoL, with survey participants who reported higher education and income scoring higher across all sub-scales of the SF-20 (Glasgow et al., 1997).Again, the results emphasize the complex interplay between physical, psychological, and social variables when assessing QoL among individuals with DM.

Of note, it must be kept in mind that all these variables were assessed using generic questionnaires, thus it is plausible that each individual interprets a certain questionnaire item differently based on their experiences and association while having a similar score. Overall, our results suggest that while there are common underlying stressors, such as the COVID-19 pandemic or other health-related and psychosocial circumstances that may lead to a systematic change in QoL for a larger group of people, individual life circumstances appear to be the primary drivers of instable QoL.

To understand the trajectories of QoL across time, previous research also aimed to identify distinct patterns. In contrast to our results, previous studies identified a maximum of five patterns by utilizing various methodological approaches and grouping trajectories based on group-level distributions such as growth mixture models or latent class growth analysis (Le Grande et al., 2006; Dunn et al., 2013; Schmitz et al., 2013; Yoo et al., 2016; Park et al., 2022). Based on the MCID to detect a clinically relevant change, our study presents descriptive findings of 19 patterns concerning changes in QoL to illustrate the extensive variety of QoL trajectories within our sample. Of note, the number of patterns is dependent on the number of time-points assessed, in our case three rather than only comparing the initial and final assessments. This is especially crucial as researchers may not be aware of the specific phase of the trajectory when assessing QoL. This relates to the frequency at which a certain variable is assessed in longitudinal data collection (Scholz, 2019) (see for example (Chevance et al., 2021) for an assessment of walking behavior across time). In relation to the original question of the manuscript, whether QoL can be considered a stable construct, the question remains how frequently QoL varies; because when using QoL as a single end-point for clinical trials one can never know at which point of the QoL oscillation the measurement takes place. Previous research has employed a classification approach driven by a model, retrospectively attempting to classify trajectories that were discovered, such as high-falling, high-stable, medium-stable, low-rising QoL (Park et al., 2022), or consistently good, steadily worsened, steadily improved, consistently bad (Yoo et al., 2016). Nevertheless, this methodology fails to represent all potential QoL patterns, which is primarily what we were interested in investigating to unveil the intricacy of individual patterns. We have solely investigated patterns through mathematical analysis to avoid concealing individual influential factors by grouping them into broader categories. Moreover, we used the MCID instead of *any* change to assess significant QoL modifications instead of relying solely on the provided QoL scores. It is noteworthy that despite latent growth models allowing for distinct trajectories among individuals, previous studies did not report these individual differences. However, our analysis indicates that individual factors play a significant role in determining patterns of QoL.

Generally, our approach indicates that future research should take into account the individual differences in QoL measured across time to truly assess well-being and effectiveness of interventions. In addition to these idiosyncratic factors, common underlying medical, social, and psychological factors have an impact on the trajectories of QoL and should therefore be targeted in interventions.

## 5 Limitations

One limitation of this study is the absence of differentiation between type 1 and type 2 DM. These two types of DM can have distinct characteristics and influences on QoL. Nevertheless, the majority of the literature examined has not shown significant variation in connection to diabetes type (Rodríguez-Almagro et al., 2018), This indicates that other factors, such as age and treatment regimen, may be more consequential determinants of QoL (Rubin and Peyrot, 1999). The data is drawn from individuals living within a community, which suggests that the sample is relatively healthy. Thus, making conclusive statements about individuals with significant health issues, such as geriatric patients, would be difficult. These analyses act as an initial indicator of the significance of individual QoL outcomes, and further testing should be conducted on different groups of PwPD.

Likewise, despite many advantages such as large sample sizes and standardized data collection procedures, the use of a multi-national panel-dataset may also introduce a certain bias towards the inclusion of comparably healthy participants. Still, SHARE counteracted this risk by utilizing purposive sampling to include a representative sample. Additionally, the use of computer-assisted interviewing helped guide participants through the questionnaires to reduce missing data; still it cannot be ruled out that missing data is more common in participants with worse mental and physical health. Due to the large sample size and the risk of introducing more bias (Jakobsen et al., 2017), we refrained from imputation-based methods, meaning that missing data were excluded from analysis.

Several variables in the analysis rely on self-reports, such as QoL, depressive symptoms, SRH and ADL, which implies that the responses provided cannot be objectified. Self-reported data poses issues, including the tendency for individuals to provide socially desirable responses and the risk of sampling bias (Devaux and Sassi, 2015). However, the utilization of self-report measures is necessary to understand a subjective construct such as QoL, which cannot be assessed objectively, and studies suggest that self-reports contain valuable data (Prince et al., 1999; Lenderink et al., 2012). Additionally, all self-report measures employed in data collection are both validated and commonly utilised. In addition, SHARE adopted a standardized, computer-assisted personal interviewing data collection procedure to alleviate social desirability response bias.

Additionally, the question remains of how to calculate a meaningful cut-off at which a change in QoL scores can be considered clinically relevant. We opted against simply using *any* change in the QOL score, as this would yield far too complex patterns that are difficult to interpret, since it is not feasible a change of 1 point in a questionnaire with up to 48 points truly represents a meaningful change in QoL. We thus used the MCID, which is based on the distribution of QoL scores in the studied population; however, other methods may be feasible as well that should be tested with respect to individual differences. Of note, for ease of interpretability, we did not differentiate between scores once they had exceeded the cut-off of 4 points for the MCID; in future studies, it might be beneficial to additionally assess the difference between smaller and very large changes in QoL, although these remain to be defined as well. In future studies, to understand in-depth at which point patients perceive a subjectively meaningful change in their QoL, analyses could be supplemented with detailed qualitative data.

DM is an illness that is commonly present in older age, therefore the use of the CASP to assess the stability of QoL already considers the specific characteristics of QoL in age. Still, to truly understand QoL in PwDM in particular, it may be beneficial to repeat the analyses with disease-specific questionnaires to incorporate DM-related features.

## 6 Conclusion

This study enhances understanding of the QoL for PwDM over a specific time frame. The QoL of PwDM shows significant stability over time on group level when assessed using traditional techniques like group comparison or regression models. However, our analysis has shown that individual factors have the highest predictive value of QoL over time. Though the effect of physical, psychological, and social factors on QoL is present, it is relatively less significant. While we did find group-level differences for mental and physical health, these were of small effect sizes, again highlighting the individual nature of QoL stability. Our results suggest that like for QoL, the characteristics of these variables may be highly subjective and interact idiosyncratically for different persons. Additional research is required to investigate the influence of individual factors in comparison to the previously dominant predictors of QoL. Healthcare professionals should take into account individual traits when creating interventions and support strategies for PwDM to improve their overall health and boost their capacity to manage their condition. Enhanced research is required to examine the long-term impacts of precise interventions and to broaden understanding in various demographics and cultural settings.

## Data availability statement

Publicly available datasets were analyzed in this study. This data can be found at: http://www.share-project.org.

## Ethics statement

The studies involving humans were approved by continuous ethics review by responsible ethics committees (University of Mannheim and Max Planck Society, Germany), as well as national ethics committees in participating countries as part of the SHARE data collection. The studies were conducted in accordance with the local legislation and institutional requirements. The participants provided their written informed consent to participate in this study.

## Author contributions

SM: Formal analysis, Methodology, Writing – original draft. KH: Writing – review & editing. HM: Writing – review & editing. TP: Conceptualization, Methodology, Writing – review & editing. AS: Conceptualization, Formal analysis, Methodology, Writing – review & editing.
