# Supplementary material for: Trajectories of quality of life in people with diabetes mellitus: results from the survey of health, ageing and retirement in Europe
Source: Front Psychol. 2024 Jan 11;14:1301530. doi: 10.3389/fpsyg.2023.1301530 (PMC10808439; doi:10.3389/fpsyg.2023.1301530)
Supplement: Supplementary file 1 [file Data_Sheet_1.docx]

**Supplement**

**Table 1. Linear Regression (backward) in PwDM.**

| **wave 5** | | | | **wave 6** | | | **wave 7** | | |
| --- | --- | --- | --- | --- | --- | --- | --- | --- | --- |
|  | **B** | **95 % CI** | **p** | **B** | **95 % CI** | **p** | **B** | **95 % CI** | **p** |
| (Constant) | 48.303 | 47.109 - 49.497 | **<.001** | 48.083 | 46.811 - 49.355 | **<.001** | 46.908 | 44.575 - 49.241 | **<.001** |
| sex | .733 | .375 - 1.090 | **<.001** | .432 | .085 - .780 | **.015** | .670 | .003 - 1.337 | **.049** |
| education | .065 | .024 - .106 | **.002** | .054 | .015 - .094 | **.007** | .123 | .047 - .199 | **.002** |
| R-UCLA | -1.021 | -1.155 - -.888 | **<.001** | -1.030 | -1.157 - -.904 | **<.001** | -1.075 | -1.311 - -.839 | **<.001** |
| memory function | -.710 | -.903 - -.517 | **<.001** | -.633 | -.816 - -.450 | **<.001** | -.744 | -1.098 - -.389 | **<.001** |
| physical inactivity | -.859 | -1.409 - -.310 | **.002** | -1.190 | -1.710 - -.670 | **<.001** | -2.130 | -3.082 - -1.178 | **<.001** |
| SRH | -1.209 | -1.438 - -.981 | **<.001** | -1.085 | -1.311 - -.859 | **<.001** | -.691 | -1.120 - -.262 | **.002** |
| EURO | -.832 | -.922 - -.743 | **<.001** | -.787 | -.876 - -.699 | **<.001** | -.892 | -1.058 - -.725 | **<.001** |
| mobility limitations | -.321 | -.410 - -.232 | **.045** | -.214 | -.307 - -.122 | **<.001** | -.357 | -.523 - -.192 | **<.001** |
| pain |  |  |  | .118 | .024 - .213 | **.014** |  |  |  |
| number of chronic diseases |  |  |  | -.122 | -.238 - -.005 | **.041** |  |  |  |
|  | adjusted R2 = 0.468, *F*(8, 2961) = 327.061, *p* < .001, Durbin-Watson = 1.647 | | | adjusted R2 = 0.469, *F*(10, 2956) = 262.805,  *p* < .001, Durbin-Watson = 1.630 | | | adjusted R2 = 0.523, *F*(8, 795) = 110.967, *p* < .001, Durbin-Watson = 1.584 | | |
| dependent variable | CASP for QoL in each wave | | | | | | | | |
| initial predictors | Gender, Current job situation, Age, Limitations with ADL, Years of education, BMI, R-UCLA Loneliness Scale, Score of memory test, Troubled with pain, Marital status, Physical inactivity, Number of chronic deseases, general health, EURO depression scale, Mobility limitations in each wave | | | | | | | | |
| final predictors | Mobility limitations, Years of education, Score of memory test, Gender, R-UCLA, Physical inactivity, general health, EURO depression scale | | | Mobility limitations, Years of education, Score of memory test, Gender, R-UCLA, Physical inactivity, general health, EURO depression scale, number of chronic diseases, pain | | | Mobility limitations, Years of education, Score of memory test, Gender, R-UCLA, Physical inactivity, general health, EURO depression scale | | |
| CASP = control, autonomy, self-realization, and pleasure QoL score; SRH = self-rated health; EURO-D = depressive symptoms questionnaire; CI = Confidence interval | | | | | | | | | |

**Table 2. Comparison between stable and unstable QoL.**

| **wave 7 PwDM** | | **Total**  **n = 2989** | **stable qol**  **n = 1034**  **(34.6 %)** | **unstable qol**  **n = 1955**  **(65.4 %)** | **U-Test R2** |
| --- | --- | --- | --- | --- | --- |
|  |  | **Median (IQR)** | **Median (IQR)** | **Median (IQR)** |  |
| Age | | 72 (66-78) | 72 (66-78) | 72 (66-79) | p = .552 |
| BMI in kg/m2 | | 29 (26-33) | 29 (26-33) | 29 (26-33) | p = .445 |
| education | | 11 (8-13) | 11 (8-14) | 11 (8-13) | **.005***** |
| limitations in ADL | | 0 (0) | 0 (0) | 0 (0) | **.003**** |
| R-UCLA | | 3 (3-5) | 3 (3-4) | 3 (3-5) | **.012**** |
| EURO | | 2 (1-4) | 2 (1-3) | 3 (1-5) | **.032***** |
| number of chronic deseases | | 3 (2-4) | 3 (2-4) | 3 (2-5) | **.002*** |
| Mobility limitations | | 2 (0-4) | 2 (0-4) | 2 (0.5) | **.004***** |
| verbal fluency | | 18 (14-23) | 19 (15-24) | 17 (13-22) | **.023***** |
|  |  | **n (%)** | **n (%)** | **n (%)** | **chi2 Cramér’s V** |
| Gender | Male | 1449 (48.5%) | 515 (49.8%) | 934 (47.8%) | p = .290 |
|  | Female | 1540 (51.5%) | 519 (50.2%) | 1021 (52.2%) |  |
|  | Missing | 0 | 0 | 0 |  |
| Marital status | Married, living with spouse | 1881 (62.9%) | 683 (66.1%) | 1198 (61.3%) | p = .171 |
|  | Registered partnership | 36 (1.2%) | 11 (1.1%) | 25 (1.3%) |  |
|  | Married, not living with spouse | 39 (1.3%) | 11 (1.1%) | 28 (1.4%) |  |
|  | Never married | 154 (5.2%) | 51 (4.9%) | 103 (5.3%) |  |
|  | Divorced | 248 (8.3%) | 84 (8.1%) | 164 (8.4%) |  |
|  | Widowed | 631 (21.1%) | 194 (18.8%) | 437 (22.4%) |  |
|  | Missing | 0 | 0 | 0 |  |
| SRH | Excellent | 34 (1.1%) | 17 (1.6%) | 17 (0.9%) | **.099***** |
|  | Very good | 194 (6.5%) | 79 (7.6%) | 115 (5.9%) |  |
|  | Good | 919 (30.7%) | 359 (34.7%) | 560 (28.6%) |  |
|  | Fair | 1272 (42.6%) | 423 (40.9%) | 849 (43.4%) |  |
|  | Poor | 570 (19.1%) | 156 (15.1%) | 414 (21.2%) |  |
|  | Missing | 0 | 0 | 0 |  |
| job situation | Retired | 2292 (77.5%) | 813 (79.1%) | 1479 (76.6%) | **.070*** |
|  | Employed or self-employed | 307 (10.4%) | 117 (11.4%) | 190 (9.8%) |  |
|  | Unemployed | 35 (1.2%) | 10 (1.0%) | 25 (1.3%) |  |
|  | Permanently sick | 98 (3.3%) | 19 (1.8%) | 79 (4.1%) |  |
|  | Homemaker | 185 (6.3%) | 57 (5.5%) | 128 (6.6%) |  |
|  | Other | 41 (1.4%) | 12 (1.2%) | 29 (1.5%) |  |
|  | Missing | 31 (1.0%) | 6 (0.6%) | 25 (1.3%) |  |
| memory function | Excellent | 45 (5.5%) | 16 (5.4%) | 29 (5.6%) | **.111*** |
|  | Very good | 119 (14.6%) | 54 (18.3%) | 65 (12.5%) |  |
|  | Good | 351 (43.0%) | 133 (45.1%) | 218 (41.8%) |  |
|  | Fair | 249 (30.5%) | 79 (26.8%) | 170 (32.6%) |  |
|  | Poor | 53 (6.5%) | 13 (4.4%) | 40 (7.7%) |  |
|  | Missing | 2172 (72.7%) | 739 (71.5%) | 1433 (73.3%) |  |
| Physical inactivity | No | 659 (80.7%) | 256 (86.8%) | 403 (77.2%) | **.116***** |
|  | Yes | 158 (19.3%) | 39 (13.2%) | 119 (22.8%) |  |
|  | Missing | 2172 (72.7%) | 739 (71.5%) | 1433 (73.3%) |  |
| pain | Yes | 471 (57.6%) | 164 (55.6%) | 307 (58.8%) | p = .371 |
|  | No | 346 (42.4%) | 131 (44.4%) | 215 (41.2%) |  |
|  | Missing | 2172 (72.7%) | 739 (71.5%) | 1433 (73.3%) |  |
| effect sizes are denoted in **bold** and indicated as statistically significant by asterisks  *** p < .001, ** p < 0.01, * p < 0.05  IQR = Interquartile range; BMI = body mass index; ADL = activites of daily living; CASP = control, autonomy, self-realization, and pleasure QoL score; EURO-D = depressive symptoms questionnaire; R-UCLA = Revised UCLA Loneliness Scale; SRH = self-rated health | | | | | |
